# Vagus nerve stimulation in treatment-resistant depression. Long-term clinical outcomes

**DOI:** 10.1192/j.eurpsy.2021.1316

**Published:** 2021-08-13

**Authors:** S. Rosson, N. Bresolin, D. D’Avella, L. Denaro, A. Landi, S. Caiolo, M. Lussignoli, T. Toffanin, G. Pigato

**Affiliations:** 1 Psychiatry Clinic, Azienda Ospedaliera di Padova, Padua, Italy; 2 Neuroscienze, U.O.C. di Neurochirurgia Pediatrica, Padova, Italy

**Keywords:** VNS, VAGUS NERVE STIMULATION, DRUG-RESISTENT DEPRESSION

## Abstract

**Introduction:**

Vagus nerve stimulation (VNS) is a neuromodulation technique approved for Treatment-Resistant Depression (TRD). Evidence regarding its long-term efficacy and safety is still scarce.

**Objectives:**

To descriptively report a case series of 3 patients undergoing adjunctive VNS for TRD with an over 10-year follow-up.

**Methods:**

We investigated outcomes of clinical interest in patients with ongoing VNS for at least 10 years after the device implantation. They had participated in a larger single-arm interventional study conducted at the University Hospital of Padua. They were diagnosed with chronic unipolar (1), recurrent unipolar (1), and bipolar (1) TRD.

**Results:**

Our 3 cases had an average 14-year history of psychiatric disease before surgery. Afterward, all subjects achieved clinical remission within two years. 2 patients experienced relapses within the first 4 years of treatment (respectively, 1 and 2 episodes). The other case showed a recurrent trend of brief relapses every two years. Only 1 individual needed to be admitted to the psychiatric unit once. None of them committed suicidal attempts. Prescription of antidepressants remained almost unchanged after the first two years. 2 individuals improved and 1 maintained their working position. Common adverse events were voice alteration (3/3), neck pain (2/3), and cough (2/3).

**Conclusions:**

Very few cases of 10-year VNS for TRD have been reported so far. For our subjects, VNS was most likely to have a major impact on the clinical course of the disease. This treatment can be a safe and effective adjunctive intervention in a subgroup of patients with TRD.

